# A Novel Bioinformatics Strategy to Analyze Microbial Big Sequence Data for Efficient Knowledge Discovery: Batch-Learning Self-Organizing Map (BLSOM)

**DOI:** 10.3390/microorganisms1010137

**Published:** 2013-11-20

**Authors:** Yuki Iwasaki, Takashi Abe, Kennosuke Wada, Yoshiko Wada, Toshimichi Ikemura

**Affiliations:** 1 Department of Bioscience, Nagahama Institute of Bio-Science and Technology, Nagahama-shi, Shiga-ken 526-0829, Japan; E-Mails: b105023@nagahama-i-bio.ac.jp (Y.I.); k_wada@nagahama-i-bio.ac.jp (K.W.); y_wada@nagahama-i-bio.ac.jp (Y.W.); t_ikemura@nagahama-i-bio.ac.jp (T.I.); 2 Japan Society for the Promotion of Science, Chiyoda-ku, Tokyo 102-0083, Japan; 3 Department of Information Engineering, Faculty of Engineering, Niigata University, Niigata-ken 950-2181, Japan; 4 Faculty of Medicine, Shiga University of Medical Science, Shiga-ken 520-2121, Japan

**Keywords:** metagenome, oligonucleotide composition, influenza virus, big data, peptide composition, bioinformatics, SOM, genome signature, microbial community

## Abstract

With the remarkable increase of genomic sequence data of microorganisms, novel tools are needed for comprehensive analyses of the big sequence data available. The self-organizing map (SOM) is an effective tool for clustering and visualizing high-dimensional data, such as oligonucleotide composition on one map. By modifying the conventional SOM, we developed batch-learning SOM (BLSOM), which allowed classification of sequence fragments (e.g., 1 kb) according to phylotypes, solely depending on oligonucleotide composition. Metagenomics studies of uncultivable microorganisms in clinical and environmental samples should allow extensive surveys of genes important in life sciences. BLSOM is most suitable for phylogenetic assignment of metagenomic sequences, because fragmental sequences can be clustered according to phylotypes, solely depending on oligonucleotide composition. We first constructed oligonucleotide BLSOMs for all available sequences from genomes of known species, and by mapping metagenomic sequences on these large-scale BLSOMs, we can predict phylotypes of individual metagenomic sequences, revealing a microbial community structure of uncultured microorganisms, including viruses. BLSOM has shown that influenza viruses isolated from humans and birds clearly differ in oligonucleotide composition. Based on this host-dependent oligonucleotide composition, we have proposed strategies for predicting directional changes of virus sequences and for surveilling potentially hazardous strains when introduced into humans from non-human sources.

## 1. Introduction

The phylogenetic analysis based on sequence homology searches is a well-established and an irreplaceably important method for studying gene and protein sequences [1,2,3]. However, it inevitably depends on alignments of sequences, which is potentially error-prone and troublesome, especially for distantly related sequences. This difficulty becomes increasingly evident as the number of sequences obtained from a wide range of species, including novel species, dramatically increases (e.g., up to millions of sequences), because of the revolutionized progress of the high-throughput DNA sequencing method. Due to this recent advancement, genomic sequence data of microorganisms, which include metagenomic sequences from clinical and environmental samples, have increased dramatically in the international DNA databanks DDBJ (DNA Data Bank of Japan, National Institute of Genetics, Shizuoka, Japan)/EMBL (European Molecular Biology Laboratory, Heidelberg, Germany)/GenBank (National Center for Biotechnology Information, Bethesda, MD, USA), and novel bioinformatics methods are needed for comprehensive analyses of the big sequence data, such as millions of sequences.

In order to establish a method to complement the sequence homology searches, we have developed an alignment-free clustering method “BLSOM” (batch-learning self-organizing map) [4,5], which can analyze millions of sequences simultaneously [6], and BLSOM has been used in many genome studies, including metagenome studies [6]. In this review, we will explain the usefulness of BLSOM by showing examples of its application to various genome and metagenome studies conducted in Japan.

## 2. An Alignment-Free Clustering Method “BLSOM” Developed for Genome Informatics

G+C% has been used as a fundamental value for phylogenetic classification of microbial genomes, including viral genomes, but G+C% is apparently too simple to differentiate a wide variety of genomes. Oligonucleotide composition, on the other hand, can be used to distinguish the species, even with the same G+C%, because the oligonucleotide composition varies significantly among the genomes and is called a “genome signature” [7]. Kohonen’s self-organizing map (SOM) is a powerful tool for clustering and visualizing high-dimensional vectorial data on a two-dimensional plane [8,9]. For codon and oligonucleotide composition handled as high-dimensional vectorial data, we have modified the conventional SOM to “BLSOM” [4,5], which is suitable for genome sequence analyses and for high-performance parallel-computing. Therefore, BLSOM can analyze big data, such as millions of genomic sequences, simultaneously [6,10,11].

In Kohonen’s original SOM, the initial vectorial data were set by random values, but in BLSOM, the initial vectors were set based on the widest scale of the sequence distribution in the oligonucleotide frequency space with PCA (principal component analysis). Weights in the first dimension (*I*) were arranged into lattices corresponding to a width of five times the standard deviation (5σ_1_) of the first principal component: the second dimension (*J*) was defined by the nearest integer greater than σ_2_/σ_1_ × *I*; and *I* was set in the present study as the average number of sequence data per neuron, becoming ten. σ_1_ and σ_2_ were the standard deviations of the first and second principal components, respectively. The weight vector on the *ij*th lattice (*w_ij_*) was represented as follows (*i* and *j* represent the position of lattice points):

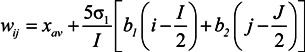
(1)
where *x_av_* is the average vector for oligonucleotide frequencies of all input vectors, and *b_1_* and *b_2_* are eigenvectors for the first and second principal components. Weight vectors (*w_ij_*) were set and updated as described previously [10]. The BLSOM program can be obtained from UNTROD Inc. (Aoyama, Nara, Japan) and from Niigata University (Niigata, Japan).

## 3. Basic Characteristics of BLSOM Separation

Since oligonucleotide composition varies significantly among species even with the same genome G+C%, the oligonucleotide composition has been used to distinguish the genome characteristics of a wide range of species and, thus, designated as a “genome signature”. To show the clustering ability of the oligonucleotide-BLSOM for a wide range of species and to explain the basic features of the BLSOM separation (self-organization), we first analyzed pentanucleotide compositions in 100-kb sequence fragments derived from ten vertebrate genomes covering a wide phylogenetic range.

**Figure 1:**
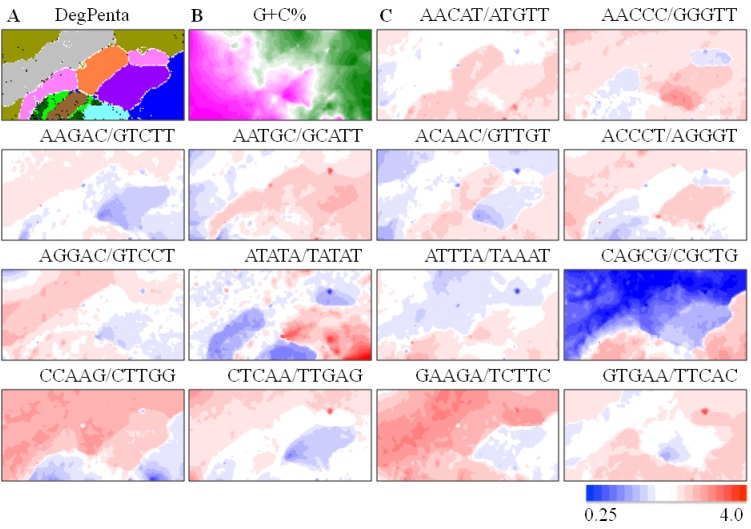
Batch-learning self-organizing map (BLSOM) with pentanucleotide compositions in 100-kb sequences from ten vertebrate genomes. (**A**) DegPenta. Lattice points containing sequences from multiple species are indicated in *black*, and those containing sequences from a single species are colored as follows: medaka (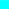), stickleback (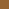), *Takifugu rubripes* (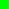), *Tetraodon nigroviridis* (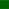), zebrafish (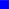), chicken (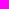), human (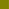), mouse (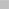), lizard (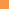) and *Xenopus* (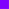). Lattice points containing no sequences after BLSOM calculation are indicated in *white blank*; (**B**) G+C%. The G+C% obtained for each lattice point on DegPenta is divided into 21 groups containing an equal number of lattice points, and the highest and lowest groups are shown in dark wine red and dark green, respectively, as presented under this panel [13]; and (**C**) Examples of pentanucleotides diagnostic for species- and phylotype-dependent separation. Occurrence of pentanucleotides for each lattice point has been calculated and normalized with the occurrence expected from the mononucleotide composition for each lattice point [11]. This observed/expected ratio is indicated in color, presented under the panel **C**.

In the DNA databanks, only one strand of complementary sequences is registered, and the strand is chosen rather arbitrarily in the registration of fragment sequences (e.g., metagenomic sequences). Our previous BLSOM analysis of a wide range of species has revealed that sequences from a single genome often give a mirror-symmetrical split on BLSOM in the vertical direction [5,12], e.g., according to the replicational direction of genomic fragments. When investigating general genome characteristics, such as the genome signature, the difference in the oligonucleotide composition between two complementary strands is not important. Furthermore, the obtained map should not be affected by the choice of strands registered in the databanks. Therefore, we constructed a BLSOM in which the frequencies of a pair of complementary oligonucleotides (e.g., AAACC and GGTTT) are summed up in each 100-kb fragment [10]. The BLSOM for this degenerate set of a pair of complementary pentanucleotides is abbreviated as DegPenta. Even without species information incorporated during BLSOM calculation, species-specific clustering (self-organization) of sequence fragments is clearly recognized (Figure 1A); similar results have been obtained on tri- and tetra-nucleotide BLSOMs (DegTri and DegTetra), as described previously [11]. On the BLSOM, lattice points containing sequences from a single species are indicated in a color specifying the species; those containing sequences from multiple species are indicated in black. Basal characteristics of BLSOM separation can be explained by Figure 1B, which shows the G+C% calculated from pentanucleotide composition at each lattice point on DegPenta, as described by Iwasaki *et al.* [13]. Sequences with higher or lower G+C% (wine red or green in Figure 1B) are located on the left or right side of the map, showing that the G+C% is reflected primarily in the horizontal direction.

The genomes of warm-blooded vertebrates are known to be composed of long-range segmental G+C% distributions designated as “isochores”, which have been connected with chromosomal bands [14,15,16,17,18]. A single genome, especially of a warm-blooded vertebrate, has a few territories on DegPenta (e.g., two chicken territories marked in pink in Figure 1A). As noted above, sequences with high or low G+C% are located on the left or right side of the map, and therefore, the split into a few sub-territories in the horizontal direction should relate, at least in part, to isochore structures.

BLSOM has an ability to visualize diagnostic oligonucleotides responsible for species-specific clustering (self-organization) and, thus, provides information concerning possible molecular mechanisms for evolutionary establishment of genome signature. For this visualization, we have previously developed a method to discover the diagnostic oligonucleotides in a way unaffected by simple differences in the G+C% of genomic sequences [6,11]; after calculating the frequency of each oligonucleotide, expect from the mononucleotide composition at each lattice point, the observed/expected ratio is indicated in red (overrepresented) or blue (underrepresented) (Figure 1C). Transitions between red and blue for various pentanucleotides often coincide exactly with borders for species-specific separations. This shows that BLSOM recognizes, in sequence fragments, a key combination of oligonucleotide frequencies that is the signature feature of each genome, and separated the sequences into species-specific territories; *i.e.*, BLSOM utilizes a complex combination of many (but not a few) oligonucleotides for the species-specific separation.

## 4. Application of BLSOM to Metagenome Studies

In the case of microorganisms, we have reported the phylotype-dependent clustering of one- and 10-kb sequences from a wide phylogenetic range of prokaryote genomes on DegTri and DegTetra [5,10]. In the present review, we will explain the BLSOM application to metagenome analyses because of the wide and current interest in microorganism research. Metagenomics analyses of uncultured microorganisms in clinical and environmental samples should allow clarifications of the microbial communality structure in an environmental sample, as well as extensive surveys of genes important in medical and environmental sciences and useful in industrial applications [19,20,21,22,23,24,25,26,27,28,29]. Traditional methods of phylogenetic assignment of metagenomic sequences have been based on sequence homology searches and, therefore, are inevitably focused on well-characterized genes, for which a set of orthologous sequences required for constructing a reliable phylogenetic tree is available. However, the well-characterized genes often are not industrially attractive. The present alignment-free clustering method, BLSOM, is the most suitable for phylogenetic assignment of all kinds of sequences, including novel gene sequences, because sequences can be phylogenetically separated, solely depending on oligonucleotide composition. It should also be noted that a large number of viral sequences have been found in metagenomic sequences [30]. Because viral genomes do not contain rRNA genes, the rDNA-sequencing method cannot be applied to clarify of the viral community structure in an environmental sample. In contrast, BLSOM can be used for viral sequences, because it can classify viral sequences according to their phylogenetic groups [13].

### 4.1. Application to Phylogenetic Classification of Millions of Metagenomic Sequences

As an example of the BLSOM application to a large-scale metagenome study, we introduce here our previous analysis [10] on metagenomic sequences obtained from the Sargasso Sea near Bermuda reported by Venter *et al.* [29]. They applied shotgun sequencing to mixed genomes collected from the Sargasso Sea and deposited approximately one million sequence fragments (a total of 1 Gb) in DDBJ/EMBL/GenBank, which must have been derived from a wide phylogenetic range of species; *i.e.*, a library with a high genome complexity. In the BLSOM analysis, we also included sequences derived from the microbe mixture in an acidophilic biofilm growing in acid mine drainage reported by Tyson *et al.* [31], as a library with a low genome complexity.

**Figure 2:**
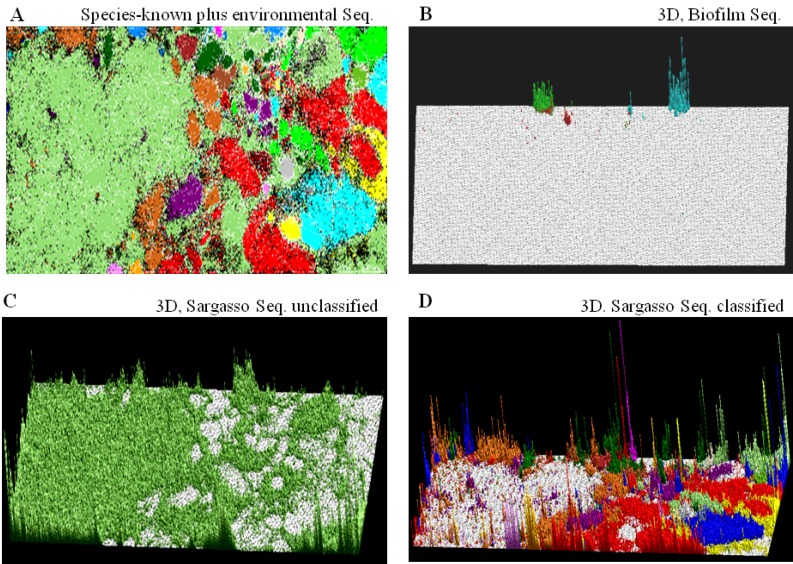
Phylogenetic classification of environmental metagenome sequences. (**A**) “Species-known plus environmental Seq.”. The degenerate tetranucleotide (DegTetra) set for species-known plus environmental sequences. The BLSOM has been constructed with 211,000 5-kb sequences from 1502 prokaryotes plus 218,000 Sargasso and 5000 biofilm 1-kb sequences; Sargasso and biofilm sequence entries longer than 1 kb were selected and divided into 1-kb fragments, and the residual segment less than 1 kb was omitted from the analysis. Lattice points containing only Sargasso or biofilm sequences are indicated in color (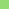) or (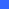); those including environmental and species-known sequences or those from more than one known phylotype are indicated in black, and those containing species-known sequences from a single phylotype are indicated in color as follows: α-proteobacteria (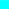), β-proteobacteria (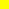), γ-proteobacteria (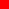), δ-proteobacteria (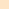), ε-proteobacteria (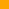), Actinobacteria (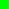), Aquificae (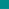), Bacteroidetes (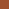), Chlamydiae (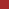), Chlorobi (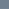), Chloroflexi (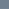), Crenarchaeota (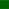), Cyanobacteria (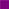), Deinococcus-Thermus (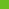), Dictyoglomi (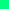), Euryarchaeota (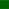), Fibrobacteres (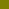), Firmicutes (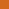), Fusobacteria (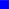), Nitrospirae (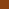), Planctomycetes (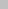), Spirochaetales (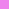), Thermodesulfobacteriales (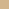), Thermotogales (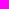) and Verrucomicrobiae (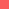); (**B**) “3D, Biofilm Seq.”. The square root of the number of biofilm sequences classified into each lattice point is shown by the height of the bar distinctively colored to show the dominant species reported by Tyson *et al.* [31]: Ferroplasma (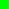), Leptospirillum (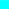) and Thermoplasmatales (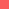); (**C**) “3D, Sargasso Seq. unclassified”. The number of Sargasso sequences located at the lattice points containing no species-known sequences is shown by the height of the bar indicated in color (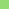), as described in **B**; and (**D**) “3D, Sargasso Seq. classified”. The number of Sargasso sequences classified into the lattice points containing species-known sequences from a single phylotype is indicated by the height of the bar distinctively colored to show the phylotype, as described in **A**. For details, see Abe *et al.* [10].

In the calculation of tetranucleotide frequencies, the paired-end reads in metagenomic sequencing, such as two 500-nt sequences from one genomic fragment, can be used as a single 1-kb sequence. Oligonucleotide frequencies in these environmental metagenome sequences were thus calculated and normalized for sequence length, and DegTetra was constructed with the oligonucleotide compositions in metagenomic sequences plus those in 1-kb sequences derived from all prokaryotic genome sequences available at that time. In the “species-known plus environmental Seq. (sequences).” (Figure 2A), lattice points that contained only Sargasso or biofilm sequences are indicated by (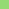) or (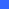), respectively; those containing sequences from a single known phylotype are colored as described in the legend; and those including sequences from more than one phylotype or including both environmental sequences and species-known sequences are shown in black; for details, see Abe *et al.* [10].

Most of the biofilm sequences derived from the low-complexity library are located in a few distinct territories in the “3D, Biofilm Seq.” panel (Figure 2B), where the number of biofilm sequences classified into each lattice point is shown by the height of one colored bar. This indicates that most sequence fragments derived from one genome in a metagenome library can be reassociated *in silico* and, thus, provides the rationale for phylogenetic classification of sequences, even those derived from a high-complexity library. The number of Sargasso sequences classified into lattice points containing no sequences from known species is indicated by the height of the (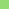) bar in the “Sargasso Seq. unclassified” panel (Figure 2C); 79% of the Sargasso sequences belonged to this phylotype-unclassified category. These Sargasso sequences should correspond to sequences derived from genomes that had been poorly-characterized at that time. The remaining 21% of the Sargasso sequences are associated with species-known sequences (black lattices in Figure 2A); the number of Sargasso sequences classified into lattice points containing species-known sequences is indicated by the height of the colored bar in the “3D, Sargasso Seq. classified” panel (Figure 2D), which represents the phylotype of the associated species-known sequences; and 92 genera, whose sequences are associated with the Sargasso sequences, have been reported together with the numbers of the associated Sargasso sequences [10].

One goal of metagenomic studies is to reconstruct multiple genomes, at least for dominant species in an environment, by sequencing a large number of fragment sequences especially derived from a low genome-complexity library. For this purpose, Banfield and her colleagues [32] have used a new type of SOM, “ESOM (emergent SOM)” [33], and successfully obtained phylotype-dependent classification of metagenomic sequences by analyzing the abovementioned acidophilic biofilm with the tetranucleotide ESOM. BLSOM can also be used for this purpose, as described later.

### 4.2. A General Strategy of Phylogenetic Assignments of Metagenomic Sequences

In the method explained in Figure 2, the metagenomic sequences that are associated (self-organized) with sequences derived from a species-known genome are considered to be derived from this or the closely related genome. In other words, the metagenomic sequences that are not clustered with species-known sequences are assigned to be derived from novel genomes poorly represented in the DNA databanks. In the case of this method, BLSOM has to be constructed for each metagenome study, and this is rather troublesome. To solve this issue, we have developed a mapping method, in which metagenomic sequences are mapped on the BLSOM that is constructed in advance with fragment sequences (e.g., 1-kb sequences) derived from all species-known genomes; once this large-scale BLSOM is constructed for all available genome sequences at a certain time, it is enough to renew the BLSOM after a significant increase of the sequences derived from new phylogenetic groups.

Since we expect phylogenetic classification of species-unknown metagenomic sequences derived from a wide range of clinical and environmental samples, the abovementioned large-scale BLSOM should be constructed with all available genome sequences, not only from species-known prokaryotes, but also from eukaryotes, as well as from viruses and organelles. When a high-performance super computer is used, such a massive amount of sequences can be clustered (self-organized) according to phylotype with high accuracy [6,11], because the BLSOM algorithm is suitable for high-level parallel computing. Once the large-scale BLSOM has been constructed, the mapping of metagenomic sequences onto this large-scale BLSOM can be conducted with PCs. This large-scale BLSOM and the program for this mapping are available from Niigata University.

**Figure 3:**
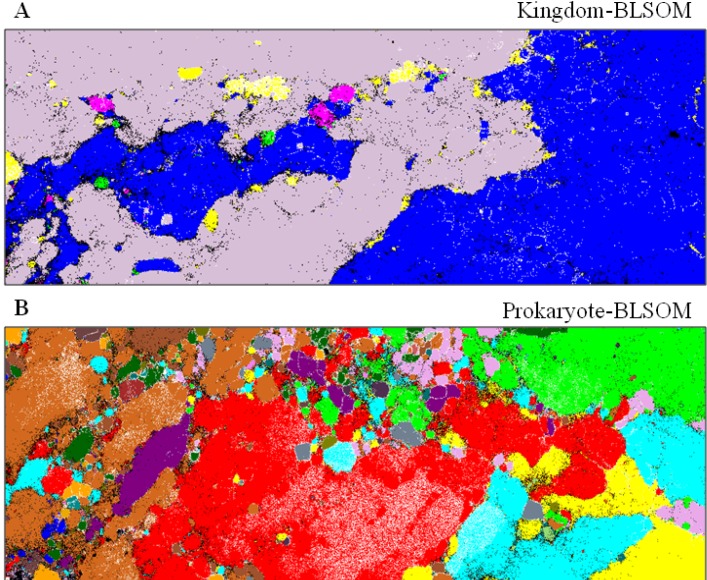
BLSOMs for phylogenetic classification of metagenomic sequences. (**A**) Kingdom-BLSOM. DegTetra has been constructed with tetranucleotide compositions in all 5-kb sequences derived from the genome sequences of 111 eukaryotes, 2813 prokaryotes,1728 mitochondria, 110 chloroplasts and 31,486 viruses. Lattice points containing sequences from multiple categories are in *black*, and those containing sequences from a single category are indicated in a color: eukaryotes, *lavender*; prokaryotes, *dark blue*; mitochondria, *pink*; chloroplasts, *green*; viruses, *yellow*; and (**B**) Prokaryote-BLSOM. DegTetra of 5-kb sequences derived from species-known prokaryotes currently available. For details, see our original paper [34].

As a general strategy to estimate the phylotypes of the metagenomic sequences obtained from a wide range of samples, we have constructed three types of BLSOMs, namely Kingdom-, Prokaryote- and Genus-BLSOM, using all genomic sequences deposited in DDBJ/EMBL/GenBank [34]. Specifically, Kingdom-BLSOM (Figure 3A) has been constructed with tetranucleotide frequencies in all 5-kb sequences derived from the whole genome sequences of 111 eukaryotes, 2813 prokaryotes, 1728 mitochondria, 110 chloroplasts and 31,486 viruses. To conduct more detailed phylotype assignments for the prokaryotic sequences that have been mapped into prokaryotic territories on the Kingdom-BLSOM, Prokaryote- and Genus-BLSOMs have been constructed with a total of 3,500,000 of 5-kb sequences from 3157 prokaryotic species, for which at least 10 kb of sequences are available from DDBJ/EMBL/GenBank.

In our standard procedure, we first map the metagenomic sequences longer than 500 bp on Kingdom-BLSOM after normalization of the sequence length, by finding the lattice point with the minimum Euclidean distance in the multidimensional space. To identify further detailed phylogenies of the metagenomic sequences that are located in prokaryotic territories on the Kingdom-BLSOM, these are successively mapped on Prokaryote-BLSOM (Figure 3B). Similar stepwise mappings of metagenomic sequences on BLSOMs that have been constructed with sequences from more detailed phylogenetic categories (e.g., phylum and genus) are continued in order to get further detailed phylogenetic information.

To evaluate the accuracy of BLSOMs for taxonomic classification of metagenomic sequences, we have previously conducted the BLSOM analysis of the simulated datasets of varying complexities [34]. When sequences longer than 300 bp were mapped, approximately 90% and 70% were correctly classified to the kingdom and phylum levels, respectively.

Because BLSOM does not require orthologous sequence sets, the present alignment-free method can provide a systematic strategy for revealing the microbial diversity and relative abundance of different phylogenetic members of uncultured microorganisms, importantly including viruses. The BLSOM method has already been used in various metagenome analyses of clinical and environmental samples conducted in Japan, as briefly explained below.

### 4.3. An Application to a Microbiome Analysis of Ticks and Other Applications

Ticks are vectors for bacterial, viral and protozoal pathogens and act not only as vectors, but also as reservoirs of tick-transmitted microbes. Because of this medical and social importance, it has become an urgent necessity to survey the bacterial pathogens of the microorganisms in ticks. To clarify the microbial gut community in various ticks, we applied BLSOM to a metagenomic study of a bacteria-enriched fraction prepared from six tick species and predicted the microbial diversity and relative abundance of microorganisms [34]. Each sample was dominated by different microbes, showing that each tick is a reservoir of specific pathogens. In addition to bacteria previously shown to be associated with human/animal diseases, such as Anaplasma, Bartonella, Borrelia, Ehrlichia, Francisella and Rickettsia, the BLSOM analysis has detected microorganisms belonging to the phylum, Chlamydiae, in some tick species. For details, see Nakao *et al.* [34].

For phylogenetic assignment for the industrially useful enzyme genes found in metagenomic libraries, we have mapped the candidate gene sequences of interest on the large-scale BLSOMs; e.g., we have assigned phylotypes of the novel xylanase genes found from human gut samples [35] and catabolic genes isolated by the substrate-induced gene-expression screening from metagenome sequences [36]. Because BLSOM for codon usage can classify protein genes of microorganisms according to phylogenetic groups [4], codon-BLSOM has been applied to phylogenetic characterization of *Pelotomaculum thermopropionicum* in anaerobic microbiota [37].

## 5. Oligonucleotide-BLSOM Applied to Studies of the Influenza Virus Genomes

Influenza viruses present a significant threat to public health, as highlighted by the recent introduction of the swine-derived pandemic, H1N1/09 [38,39,40], into human populations. Influenza virus pandemics have been often initiated by the introduction of viruses from non-human sources followed by adaptation among humans through human-to-human transmission. One important issue in studies of viral genomes, particularly those of the influenza virus, is to predict possible changes in genomic sequence occurring in the near future [41,42]. In this review, we explain our recent finding [13,43] that BLSOM can predict the directional change of influenza A genome sequences after invasion into human populations from non-human sources, at least in a specific aspect, and, therefore, can systematically survey potentially hazardous non-human strains when introduced into human populations.

### 5.1. Host-Dependent Clustering of Influenza Virus Genome Sequences

**Figure 4:**
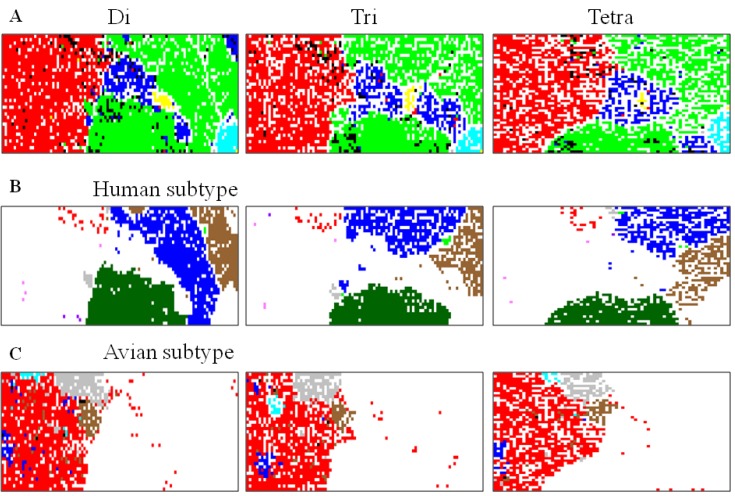
BLSOMs for influenza virus genome sequences. (**A**) Di, Tri and Tetra. Di-, tri- and tetra-nucleotide BLSOMs have been constructed for 14,447 strains of influenza A and B virus. Lattice points containing sequences from strains isolated from more than one host are indicated in black, and those containing sequences from one host are indicated in a color: avian, *red*; human, *green*; swine, *blue*; equine, *yellow*; bat, *gray*; and influenza B, *light blue*; (**B**) Human subtype. On each BLSOM in **A**, individual human virus subtypes are specified in a color representing the subtype: H1N1, *brown*; H1N1/09, *dark green*; H2N2, *gray*; H3N2, *blue*; H5N1, *red*; H7N9, *purple*; other H7 and H9, *pink*; other human subtype, *green*; and territories of other hosts are *achromatic*; and (**C**) Avian subtype. On each BLSOM in **A**, individual avian virus subtypes are specified in a color representing the subtype: H5N1, *gray*; H7N2, *light blue*; H7N3, *blue*; H9N2, *brown*; other avian subtype, *red*; and territories of other hosts are *achromatic*.

Influenza A and B virus genomes are composed of eight segments, each of which encodes primarily one or two proteins. When we include only those whose sequences are available from all eight segments, genomic sequences are available from approximately 14,000 strains of influenza viruses in the Influenza Virus Resource [44] at NCBI (National Center for Biotechnology Information, U.S. National Library of Medicine, Bethesda, MD, USA). Oligonucleotide frequencies in the eight segments are summed up for each strain, and we have constructed di-, tri- and tetra-nucleotide BLSOMs (abbreviated as Di, Tri and Tetra) for all influenza A and B strains (Figure 4). The direct target of natural selection is a virion containing a full set of the eight segments, and this genome-level BLSOM analysis should provide valuable, novel information about genome characteristics of individual strains. Influenza virus possesses the negative-sense single-stranded RNA genome, and the sequences corresponding to the coding strand are registered in the databank and, thus, are analyzed in this study; a pair of complementary oligonucleotides are not added, because the complimentary strands have clearly different biological functions. When we consider the results for the RNA genome itself, we have to make the exchange between A and U and between C and G.

In Figure 4A, lattice points containing virus strains isolated from one host species are indicated in a color representing the host, and those containing strains isolated from more than one host are in black. Though only oligonucleotide compositions have been given during the BLSOM calculation, viral sequences are clustered (self-organized) according to host; for details, see our original papers [13,43,45]. This host-dependent clustering of viral sequences should reflect the host dependency of the viral growth. Viruses are inevitably dependent on many host factors for their growth (e.g., pools of nucleotides, amino acids and tRNAs) and, at the same time, have to escape from antiviral host mechanisms, such as antibodies, cytotoxic T-cells, interferons and RNA interferences [46,47,48,49].

In Figure 4B, lattice points that contain human influenza A viruses of one subtype are specified with one color representing the subtype on each BLSOM. Among the 14,000 strains analyzed, approximately 3000 strains correspond to the pandemic H1N1 strain (H1N1/09) (dark green in Figure 4B), which has started its pandemics among humans around since April 2009. Although its origin is derived from avian strains, it has been infected to humans via swine after multiple reassortments of genome segments. Interestingly, on BLSOM, they are located apart from seasonal human H1N1 or H3N2 strains (brown or blue in Figure 4B) and surrounded by avian and swine territories (red and blue in Figure 4A and blank in Figure 4B), indicating that these H1N1/09 strains have not yet been best suited to growth in human cellular environments.

In contrast to H1N1/09 (dark green), most human H5N1 strains (red in Figure 4B and mainly black in Figure 4A) and human H7N9 strains (purple in Figure 4B and mainly black in Figure 4A) are rather scattered within the avian territory (red in Figure 4A and blank in Figure 4B). This is consistent to the view that the human H5N1 and H7N9 strains have jumped to humans, but not yet been able to spread from human to human [48,50], and therefore, they have characteristics of avian viruses. These strains are more separated from the human seasonal flu territories than H1N1/09 strains, and this difference from H1N1/09 may relate to their lower infection power in human populations. It should also be noted that a very minor portion of avian H5N1 and H9N2 strains (gray or brown in Figure 4C) are located near the human territories, showing that these strains should have oligonucleotide composition with a higher similarity to human strains than the human H5N1 and H7N9 strains already known, and these will be mentioned later in connection with potentially hazardous strains; for details, see Iwasaki *et al.* [43]. There are also the avian strains that are scattered primarily within the swine territories (red in Figure 4C). These avian strains most likely have jumped to avians from swines because these have the swine-type oligonucleotide composition.

In the case of influenza B strains (light blue in Figure 4A and blank in Figure 4B), which have repeatedly caused epidemics only among humans, they form a territory more distant from the avian territory than other human seasonal strains.

### 5.2. Retrospective Time Series Changes Visualized for Human Viruses

**Figure 5:**
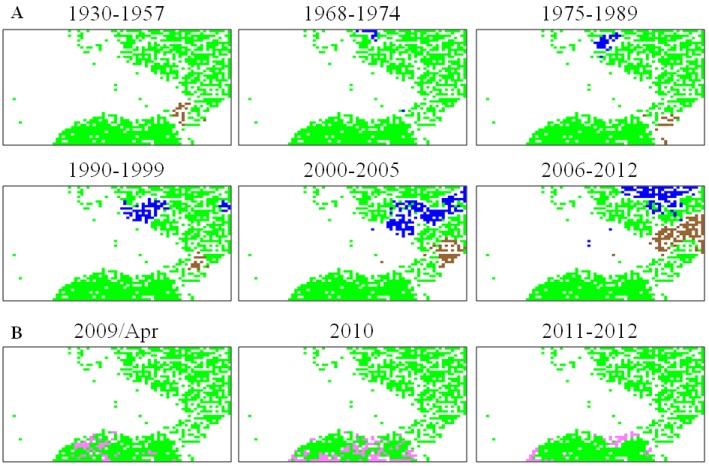
Chronological changes observed for seasonal human flu subtypes. (**A**) On Tetra listed in Figure 4A, seasonal human H1N1 and H3N2 strains isolated in different periods are separately marked in *brown* and *blue*, respectively; and (**B**) H1N1/09 strains that are isolated in different periods are separately marked in *pink*.

The prediction of genomic sequence changes in the near future is one important issue for bioinformatics studies of influenza viruses. Invader viruses will change their genome sequences on a balance between stochastic processes of mutation and selective forces derived from various constraints, including those from a new host. In other words, a certain level of change may be predictable after invasion into a new host, at least in regard to specific aspects. To examine possible directional changes, we next visualize retrospective time series changes of human seasonal H1N1 and H3N2 strains on Tetra (Figure 5A). Human seasonal H1N1 and H3N2 strains isolated in a specific time period are indicated in brown and blue, respectively; other human strains are left in green, and strains isolated from other hosts are left achromatic. Seasonal human strains isolated in a very early period of their pandemic (“1930–1957” for H1N1 and “1968–1974” for H3N2) are located near the avian territory (achromatic in Figure 4B and red in Figure 4A), and pandemic descendants isolated in later periods moved apart from the avian territory, showing time-series directional changes [43]. Figure 5B similarly visualizes time series changes of H1N1/09 strains on Tetra; strains isolated in a specific time period are indicated in pink. The major portion of the strains isolated in April 2009 are located in the vicinity of avian and swine territories, but those isolated after 2009 are primarily located near the human seasonal flu territory and, thus, apart from the avian territory; for details, see Iwasaki *et al.* [43,45]. These directional changes in the oligonucleotide composition for human pandemic strains have been confirmed by a numerical analysis of the multidimensional vectorial data [45].

Taken together, BLSOM can visualize any category of strain, in which experimental and medical research groups will be interested; e.g., visualizing strains isolated in a specific time period and/or in a specific land area, along with the characteristics of their oligonucleotide composition. This ability of BLSOM is particularly useful for the efficient survey of potentially hazardous strains in a vast number of strains isolated.

### 5.3. Diagnostic Oligonucleotides Responsible for Host-Dependent Separation

**Figure 6:**
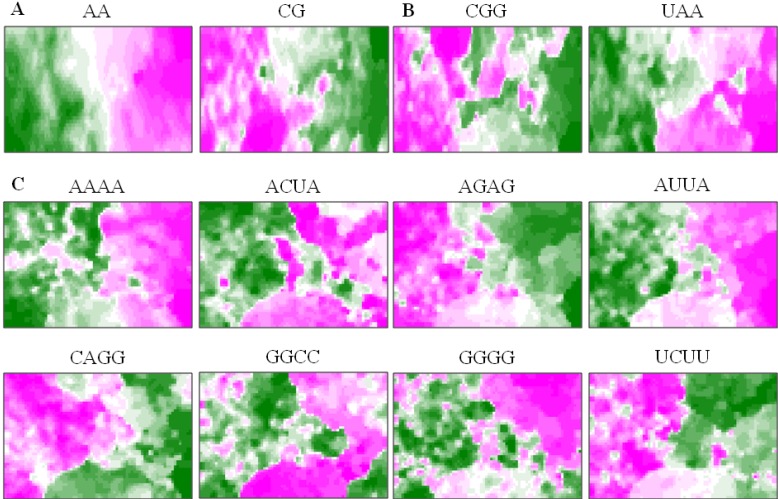
Diagnostic di-, tri- and tetra-nucleotides for host-dependent separation. (**A**,**B**) On Di and Tri listed in Figure 4A, the occurrence levels of two di- and tri-nucleotide diagnostics for host-dependent separation are indicated with different levels of two colors: *pink* (high), *green* (low) and *achromatic* (intermediate), as described by Iwasaki *et al.* [13]. (**C**) On Tetra listed in Figure 4A, the occurrences of eight tetranucleotide diagnostics for host-dependent separation are indicated, as described in (**A**,**B**).

BLSOM provides a powerful ability to visualize diagnostic oligonucleotides, which are responsible for the host-dependent clustering in this case. In order to search for the oligonucleotides that may relate to host adaptation, we have identified diagnostic oligonucleotides for the host-dependent separation on each BLSOM. In Figure 6, two examples of diagnostic di- and tri-nucleotides and eight examples of diagnostic tetranucleotides for the host-dependent separation are presented. A clear tendency is apparent; A- and U-rich oligonucleotides are preferred in human strains rather than in avian strains (e.g., AA, UAA, AAAA and AUUA); G- and C-rich oligonucleotides are preferred in avian strains rather than in human strains (e.g., CG, CGG and CAGG). This G+C% effect was previously reported by Rabadan *et al.* [51]. However, GGCC and GGGG, which are composed only of G and/or C, are preferred in human strains rather than in avian strains, and UCUU, a tetranucleotide rich in U, is preferred mainly in the avian territory. These characteristic tetranucleotide compositions, which cannot be explained by the G+C% effect, are interesting from the view of their biological significance, and therefore, detailed analyses of these oligonucleotides are in progress.

### 5.4. A Strategy for Finding Potentially Hazardous Strains

Another important issue for bioinformatics studies of influenza viruses is the search for strains that will become hazardous in the near future. Our previous papers have proposed a novel strategy for finding the potentially hazardous avian strain [43,45]. In the present review, we introduce a similar, but slightly modified strategy. Specifically, we here suppose that the seasonal human H2N2, H3N2 and H1N1/09 strains isolated at a very early stage (*i.e.*, those isolated in 1957, 1968 and April 2009, respectively) may have such characteristics that potentially prepare them for efficient human-human transmission; *i.e.*, a significant level of human-type preference. We thus focus on specific tetranucleotides, whose occurrences in these specified human strains are distinct from those in most avian strains (Table 1).

**Table 1:** Diagnostic tetranucleotides preferred in human strains isolated in the very early period.

Class	Diagnostic Tetranucleotides
High	AAGU, ACUA, ACUU, AUGA, AUUA,
	AUUU, CUAA, CUUU, GCCG, GGCC,
	UAAG, UAUC, UCAU, UUAA, UUAU
Low	ACCG, ACGC, AUCU, CUCA, CUGA,
	GAGC, GAGG, GAUC, GCAG, GCUG, GGAG

Table 2 lists avian strains with a high level of the abovementioned human-type preference. Two H1N1 strains isolated from turkeys in Ontario in 2009 have these preferences for 18 and 17 tetranucleotides out of a total of 26 diagnostic tetranucleotides listed in Table 1; designated here as a score of 18 and 17 points. These avian strains are known to be human-to-bird transmitted H1N1/09 by phylogenetic tree analysis [52]. Two H1N1 strains isolated from turkeys in the U.S. scored 17 points and are located within a swine territory near a border to the human territory, indicating swine-to-bird transmission. These findings support the suitability of the choice of the diagnostic tetranucleotides. Importantly, an H4N2 strain isolated from Pekin duck in California also has a very high score equivalent to the abovementioned human-to-bird transmitted strains, though the H4N2 subtype has not caused epidemics among humans. When avian strains with characteristics similar to this Pekin duck strain invade to humans, this may cause human-to-human transmission with a significant probability, and therefore, have a high risk potential. H4N8, H3N8, H5N2 and H6N2 strains isolated from various birds in various places also have relatively high scores, although these subtypes also have not caused epidemics in human populations (listed in bold in Table 2). In contrast, all known human H5N1 strains, which have not caused epidemics in human populations, have low scores (≤5); an avian H5N1 strain isolated from chicken in West Bengal has a higher score (seven points) than all known human H5N1 strains, indicating that this avian H5N1 strain may have a higher possibility of human-human transmission than the known human H5N1 strains. By combining mutually independent bioinformatics methods, we can develop a strategy for efficient and systematic surveillance of potentially hazardous strains that may cause new pandemics in a high probability among humans in the near future.

**Table 2:** Potentially hazardous avian strains.

Point	Subtype	Year	Strain
18	H1N1	2009	A/turkey/Ontario/FAV110
18	H1N1	1988	A/turkey/NC/19762
18	H1N1	1988	A/turkey/NC/17026
17	H1N1	2009	A/turkey/Ontario/FAV114-17
17	H1N1	1992	A/turkey/IA/21089-3
17	H1N1	1991	A/chicken/PA/35154
15	H1N1	1980	A/turkey/Kansas/4880
14	H4N2	2006	A/Pekin duck/California/P30
11	H3N8	1987	A/duck/LA/17G
11	H3N2	2011	A/turkey/Ontario/FAV-9
11	H1N2	2001	A/duck/NC/91347
10	H3N2	2011	A/turkey/Ontario/FAV-10
9	H6N2	2004	A/chicken/CA/S0403106
9	H6N2	2002	A/wild duck/Shantou/867
9	H5N2	2012	A/chicken/Taiwan/A1997
9	H5N2	2002	A/chicken/Guatemala/194573

We first calculated the average occurrence of each tetranucleotide in human H2N2, H3N2 and H1N1/09 strains isolated in the very early stage of their pandemics and then selected the tetranucleotides, for which each of the abovementioned three averages was higher or lower than the occurrences of more than 80% of the avian strains. This selection was based on the assumption that a limited portion of avian strains may have a human-type preference for some tetranucleotides. Fifteen higher and 11 lower cases of the diagnostic tetranucleotides were thus selected; for details, see Iwasaki *et al.* [43].

The avian strains that have been thought to be transferred directly from humans or swines are indicated in italic. Other avian subtype strains that have not caused pandemics among humans, and, thus, are suspected to be the potentially hazardous strains, are indicated in bold.

### 5.5. BLSOM Analyses of Individual Segments

In the analyses listed in Figure 4, Figure 5 and Figure 6, oligonucleotide frequencies in the eight segments were summed up for each strain, and BLSOMs were constructed with the summed frequencies. It should be noted here that, at the onset of a new pandemic, reassortment of virus genome segments in a certain host (e.g., swine) and successive invasion of the new reassortant into human populations were often observed. Therefore, separate analyses of eight segments are undoubtedly important: the gene-level analysis. In our previous study [13], oligonucleotide compositions of eight segments also were separately analyzed, and this showed clear host-dependent clustering of each segment on oligonucleotide BLSOMs, even though the length of the shortest segment (Segment 8) is approximately 0.8 kb. The phylogenetic relationships among sequences of individual segments found by this gene-level BLSOM were primarily consistent with those obtained with the phylogenetic tree analyses of individual genes [13].

## 6. Other Applications of BLSOM and Future Prospects

In the present review, we have introduced oligonucleotide BLSOMs applied to the genomic sequence analyses of microorganisms. In the final part of this review, we briefly explain additional BLSOM methods that have been applied to a few specific topics at the present moment, but that appear to be widely applicable to a wide range of microorganism research.

### 6.1. Addition of Computer-Generated Random Sequences

Oligonucleotide-BLSOM can be used even for the study of sequences derived from one genome. In such a study, the addition of computer-generated random sequences to real genomic sequences can successfully classify real sequences into several groups that have different oligonucleotide compositions often existing in one genome. This is because these groups of real genomic sequences tend to be surrounded by zones formed with random sequences and, thus, clearly separated from each other [12,53]. This can unveil hidden genome characteristics that are difficult to discover by other bioinformatics methods. For example, we have recently found the notable clustering of transcription-factor binding-motifs in human pericentric regions, by analyzing human genomic sequences plus computer-generated random sequences with DegPenta [12].

In the case of one microorganism genome, the addition of random sequences should separate the horizontally transferred sequences from other regular genomic sequences, on the basis of their differential oligonucleotide compositions [5]. When we added computer-generated random sequences to metagenomic sequences derived from an environmental sample, the metagenomic sequences have formed many separate regions, which are surround by zones formed by random sequences [54]. Since these separate regions have different oligonucleotide compositions from each other, these regions should represent sequences belonging to distinct phylogenetic groups. The separation of metagenomic sequences according to phylotype should provide a useful strategy to construct a nearly full sequence of one genome, using a vast amount of metagenomic sequences. This should be especially useful for a novel genome, for which a template genome for mapping is not available and, thus, the *de novo* assembling is inevitable.

### 6.2. Peptide-BLSOM for Functional Prediction of Poorly-Characterized Protein Candidates

As the result of decoding a massive amount of genomic sequences, including metagenomic sequences, a large number of protein gene candidates whose function cannot be estimated by the amino-acid sequence homology search have been progressively accumulating in international DNA databanks and, thus, remain practically of no use to science and industry. A method to predict protein functions that does not depend on the sequence homology search is an urgent need.

We have attempted to develop a BLSOM method that can predict protein functions on the basis of the similarity in oligopeptide compositions [55]. In that study, focusing on the di-, tri- and tetra-peptide compositions in the 110,000 proteins that had been classified into 2853 function-known COGs (Clusters of Orthologous Groups of proteins), we have found BLSOM conditions that faithfully reproduce the COG classification and have applied the peptide-BLSOM to predict the functions of protein candidates obtained from environmental metagenomic sequences [55]. The peptide-BLSOM has recently been used to characterize protein sequence diversity in the enzymes related to secondary metabolic pathways in plants [56]. When compared to the oligonucleotide-BLSOM, however, the peptide-BLSOM is quite a ways from methodological establishment at the present moment. Therefore, we have continued to improve the peptide-BLSOM, because this method should contribute significantly to the characterization of a vast amount of function-unknown, poorly-characterized proteins of microorganisms.
